# Inhibition of yes‐associated protein down‐regulates PD‐L1 (CD274) expression in human malignant pleural mesothelioma

**DOI:** 10.1111/jcmm.13593

**Published:** 2018-03-24

**Authors:** Ping‐Chih Hsu, Jinbai Miao, Yu‐Cheng Wang, Wen‐Qian Zhang, Yi‐Lin Yang, Chih‐Wei Wang, Cheng‐Ta Yang, Zhen Huang, Joanna You, Zhidong Xu, David M. Jablons, Liang You

**Affiliations:** ^1^ Department of Surgery Helen Diller Family Comprehensive Cancer Center University of California, San Francisco San Francisco CA USA; ^2^ Department of Thoracic Medicine Chang Gung Memorial Hospital, Linkou Taoyuan Taiwan; ^3^ Department of Thoracic Surgery Beijing Chao‐Yang Hospital Affiliated with Capital Medical University Beijing China; ^4^ Department of Pathology Chang Gung Memorial Hospital, Linkou Taoyuan Taiwan; ^5^ Department of Hepatobiliary Surgery National Cancer Center/Cancer Hospital Chinese Academy of Medical Sciences and Peking Union Medical College Beijing China

**Keywords:** checkpoint immunotherapy, hippo signaling, malignant pleural mesothelioma, PD‐L1, yes‐associated protein

## Abstract

Although tumour PD‐L1 (CD274) expression had been used as a predictive biomarker in checkpoint immunotherapy targeting the PD1/PD‐L1 axis in various cancers, the regulation of PD‐L1 (CD274) expression is unclear. Yes‐associated protein (YAP), an important oncogenic protein in Hippo signalling pathway, reportedly promotes cancer development. We investigated whether inhibition of YAP down‐regulates PD‐L1 (CD274) in human malignant pleural mesothelioma (MPM). Western blotting showed that 2 human MPM cell lines (H2052 and 211H) had increased PD‐L1 protein expression compared to H290, MS‐1 and H28 cells. In H2052 and 211H cells, PD‐L1 mRNA expression was significantly increased compared to other MPM cell lines; YAP knockdown by small interfering RNA decreased PD‐L1 protein and mRNA expression. Forced overexpression of the YAP gene increased PD‐L1 protein expression in H2452 cells. Chromatin immunoprecipitation (ChIP) assay showed the precipitation of PD‐L1 enhancer region encompassing 2 putative YAP‐TEAD‐binding sites in H2052 cells. We found that, in human MPM tissue microarray samples, YAP and PD‐L1 concurrently expressed in immunohistochemistry stain (n = 70, *P* < .05, chi‐square). We conclude that PD‐L1 is correlated with YAP expression, and inhibition of YAP down‐regulates PD‐L1 expression in human MPM. Further study of how YAP regulates PD‐L1 in MPM is warranted.

## 1 INTRODUCTION

Malignant pleural mesothelioma (MPM) is a very aggressive cancer that arises from the pleura, and most patients were diagnosed at advanced and unresectable stage.^1^ Advanced MPM has poor prognosis with a median survival about 12 months, and the treatment option for advanced MPM is very limited.^1^, ^2^

Because some cancers, including MPM, engage immune checkpoints to escape antitumour immune responses, checkpoint molecule blockade has been pursued as a therapeutic anticancer strategy.^3^, ^4^, ^5^ Immunotherapy targeting the immune checkpoint programmed death‐1 (PD‐1)/PD‐ligand (L) 1 axis has been used to treat various cancers including non‐small‐cell lung cancer, melanoma, renal cell carcinoma and Hodgkin lymphoma.^6^, ^7^, ^8^, ^9^, ^10^ Recently, PD‐1/PD‐L1 inhibitors were also used to treat patients with advanced MPM, and several clinical trials are ongoing.^11^, ^12^ Tumour PD‐L1 expression has been used as a predictive biomarker for PD‐1/PD‐L1 inhibitors.^6^, ^12^, ^13^ However, how the tumour PD‐L1 expression is regulated in cancer cells is not obvious.

Yes‐associated protein (YAP) is a key oncogenic mediator in the Hippo signalling pathway and promotes tumorigenesis, invasion, metastasis and drug resistance in several cancers.^14^, ^15^, ^16^, ^17^, ^18^ YAP has also been identified in human MPM and is correlated with tumorigenesis and cancer development, suggesting YAP is a therapeutic target for treating advanced unresectable MPM.^19^, ^20^, ^21^ YAP regulates tumour‐associated immune cells such as myeloid‐derived suppressor cells (MDSCs) and tumour‐associated macrophages (TAMs).^22^, ^23^ YAP may be involved in the immune checkpoint.^22^, ^23^ Here, we sought to investigate whether YAP is involved in the regulation of PD‐L1 expression of human MPM and whether inhibition of YAP down‐regulates PD‐L1 expression in human MPM.

## 2 MATERIALS AND METHODS

### 2.1 Cell lines and cell culture

Human mesothelioma cell lines H2052, 211H, H28 and H2452 were purchased from American Type Culture Collections (ATCC, Manassas, VA, USA), and H290 and MS‐1 were purchased from NIH (Frederick, MD, USA). Human non‐small‐cell lung cancer cell line A549 was purchased from American Type Culture Collections (ATCC). Human normal mesothelial cell line LP‐9 was purchased from the Cell Culture Core Facility at Harvard University (Boston, MA, USA). The 5 mesothelioma cell lines and A549 cells were maintained in RPMI‐1640 medium supplemented with 10% heat‐inactivated foetal bovine serum (FBS) and penicillin (100 IU/mL). LP‐9 was maintained in M199 supplemented with 15% heat‐inactivated FBS, 10 ng/mL EGF, 0.4 μg/mL hydrocortisone and penicillin (100 IU/mL). All cell lines were incubated at 37°C in a humid incubator with 5% CO_2_.

### 2.2 Tissue samples and immunohistochemistry

Fresh primary MPM and adjacent normal pleural tissues were obtained from 70 patients with MPM whose primary tumours were surgically resected. There are few exception normal pleural tissues used as normal control from non‐tumour patients on the same array. Primary human MPM samples and normal pleural samples were fixed in formalin and embedded in paraffin in 4‐μm tissue microarray sections. All human tissue samples were obtained and analysed in accordance with procedures approved by the institutional review board of the University of California, San Francisco (IRB H8714–22 942–01).

Rabbit anti‐YAP antibody (Cell Signaling, 4912; 1:500) and rabbit anti‐PD‐L1 antibody (Cell Signaling, 13684; 1:500) were used for immunohistochemistry (IHC) staining. The tissue microarray slides were deparaffinized and rehydrated and then immersed in boiled 10 mmol/L sodium citrate buffer (pH 6.0) for 10 minutes. After cooling down, slides were incubated in 3% hydrogen peroxide for 10 minutes and washed in PBS 3 times for 5 minutes. Slides were incubated with 10% normal goat serum in PBS for 30 minutes. After being washed in PBS 3 times, the slides were incubated overnight at 4°C with rabbit anti‐YAP antibody (Cell Signaling, 4912; 1:500) and rabbit anti‐PD‐L1 antibody (Cell Signaling, 13684; 1:500). The next day, the slides were incubated with biotin‐labelled secondary antibodies and streptavidin‐peroxidase (1:30) for 20 minutes each. Slides were stained for 5 minutes with 0.05% 3,3′‐diaminobenzidine tetrahydrochloride freshly prepared in 0.05 mol/L Tris‐HCl buffer (pH 7.6) containing 0.024% hydrogen peroxidase. The slides finally were counterstained with haematoxylin, dehydrated and mounted in Diatex.

The following scoring system was used: −, no stain; +, weak staining (≥10% and <30% stained cellularity considered as positive); ++, moderate staining (≥30% and <50% stained cellularity considered as positive); +++, strong staining (50% or above stained cellularity considered as positive). All scoring was carried out under a low power objective lens (10X) with a Zeiss Axioskop 2 microscope (Carl Zeiss Inc, Germany). Images were taken under 10× or 40× objective lens.

### 2.3 Small interfering RNA (siRNA) and cDNA plasmid transfection

The SMARTpool siRNA targeting YAP (YAP siRNA‐1) and a non‐targeting siRNA used as control were purchased from Thermo Scientific Dharmacon (Pittsburgh, PA, USA). Another YAP siRNA (AM16708) targeting the 3′UTR end of the YAP gene (YAP siRNA‐2) was purchased from Life Technologies (Grand Island, NY, USA). The YAP plasmid DNA (pcDNA4/HisMaxB‐YAP) used to overexpress the YAP gene in the cells was purchased from Addgene (Cambridge, MA, USA).

The mesothelioma cells were plated in 6‐well plates (for Western blot or PCR) or 24‐well plates (for reporter assay) 24 hours before transfection. Cells were transfected with 100 nmol/L of siRNA using Lipofectamine RNAiMAX (Invitrogen, Carlsbad, CA, USA). For the YAP rescue procedure with YAP forced overexpression, cells cultured in 6‐well plates were transfected with 4 μg/well of YAP plasmid DNA, and those in 24‐well plates were transfected with 0.8 μg/well. After transfection for 48 hours, cells were harvested for further analysis.

### 2.4 Western blot analysis

The total amount of protein for each sample was 20 μg. The samples were run on 4%‐20% gradient SDS‐polyacrylamide gels (Bio‐Rad Laboratories, Inc., Hercules, CA, USA) and then were transferred to Immobilon‐P nitrocellulose membranes (Millipore, Billerica, MA, USA). The membranes were probed with rabbit anti‐YAP (Cell Signaling, 4912; 1:1000), rabbit anti‐phospho Yap Ser127 (Cell Signaling, 9411; 1:1000), rabbit anti‐PD‐L1 antibody (Cell Signaling, 13684; 1:1000) and mouse anti‐GAPDH (Sigma‐Aldrich, 100242‐MM05; 1: 10 000) in 4°C overnight after being blocked with 5% non‐fat milk. The membranes were then incubated with species‐specific conjugated secondary antibodies (GE Dharmacon) at room temperature for 1 hour. An ECL blotting analysis system (Amersham Pharmacia Biotech, Piscataway, NJ, USA) was used for detecting protein expression.

### 2.5 RNA isolation, cDNA synthesis and quantitative real‐time RT‐PCR

Total RNA was extracted from cells using the High Pure RNA Isolation Kit (Roche, Indianapolis, IN, USA). RNA was transcribed to the cDNA using the iScript cDNA Synthesis Kit (Bio‐Rad), and the cDNA was used as the template for real‐time PCR. Primers and probe sequences used to detect YAP, CD274, AREG, CTGF and endogenous control gene b‐glucuronidase (GUSB) gene expression were purchased from Life Technologies (Carlsbad, CA). Real‐time PCR detection was carried out using TaqMan Technology on an Applied Biosystems 7000 sequence detection system (Applied Biosystems). Relative Quantification Software (Applied Biosystems) was used for mRNA expression analysis.

### 2.6 Luciferase reporter assay

The 8× GTIIC‐luciferase plasmid (Addgene) and Renilla luciferase pRL‐TK plasmid (Promega, Madison, WI, USA) were cotransfected into cell lines using the transfection reagent Lipofectamine 2000 (Invitrogen). After 48 hours, cells were lysed and the lysate was transferred into a 96‐well plate for analysis using the Dual‐Luciferase Reporter Assay Kit (Promega). Luminescent signalling was measured on a GloMax‐96 Microplate Luminometer (Promega) according to the manufacturer's instructions.

### 2.7 ChIP assay

Fragmented chromatin from H2052 cells was incubated with anti‐IgG (negative control), anti‐YAP and anti‐POL‐II (positive control). Recruited DNA was subjected to PCR using the primers for distal enhancer regions of PD‐L1, and PCR products were electrophoresed in agarose gel. The ChIP assay was conducted using the Chromatin Immunoprecipitation (ChIP) Assay Kit (Millipore Corporation). Polyclonal antibodies for YAP (Cell Signaling Technology) and control rabbit antibody for IgG (Cell Signaling Technology) were used for ChIP. Two‐pair primers were used for RT‐PCR to amplify the PD‐L1 gene. One pair consisted of 5′‐TCGGTCTGTGAAGGACTGC‐3′ and 5′‐ACCGTTGAGGAATGGATGAA‐3′, resulting in a product size of 203 bp, and the other consisted of 5′‐CCACCACCATTATCTAATTCCA‐3′and 5′‐AAGGAGCCAGACACAAAAGG‐3′, resulting in a product size of 210 bp.

### 2.8 Cell proliferation assay

Mesothelioma cell lines 211H, H2452, H290 and H2052 were cultured in a 96‐well plate. The cell count placed in each well was 3300. Cells were harvested at the time‐point of 0, 24, 48 and 72 hours. Cells were lysed, and CellTiter‐Glo Luminescent Cell Viability Assay reagent (Promega) was added to generate luminescent signalling. Luminescent signalling was detected using the GloMax‐96 Microplate Luminometer. Proportional cell proliferation was analysed with GraphPad Prism 5.0 software (GraphPad Software, Inc., La Jolla, CA, USA).

### 2.9 Statistical analysis

Data are presented as mean ± standard deviation (SD) from 3 independent experiments. All statistical analyses were performed with GraphPad Prism (version 6.0; GraphPad Software, San Diego, CA, USA). One‐way ANOVA followed by Scheffe multiple comparisons were used to compare the differences among multiple groups. A significant difference was considered when the *P* value from a 2‐tailed test was <.05.

## 3 RESULTS

### 3.1 PD‐L1 (CD274) and YAP expression in malignant pleural mesothelioma cell lines

**Figure 1:**
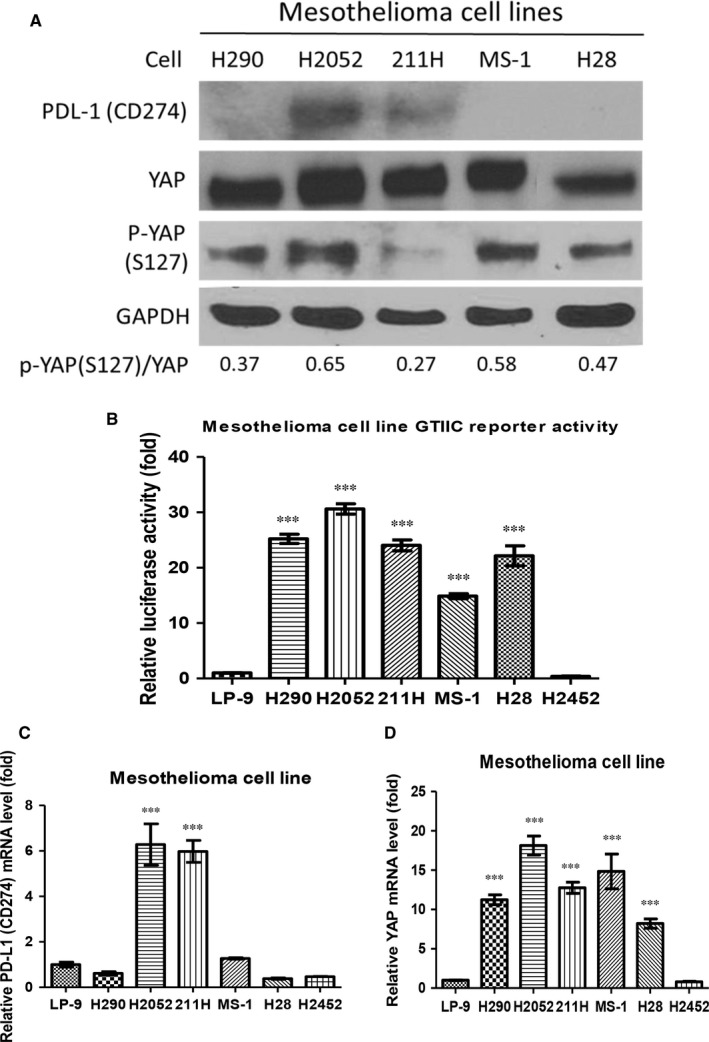
PD‐L1 and yes‐associated protein (YAP) expression in human malignant pleural mesothelioma cell lines. (A) PD‐L1, YAP and p‐YAP (S127) protein expression and p‐YAP (S127)/YAP ratio in mesothelioma cell lines. (B) GTIIC reporter activity of human normal mesothelial cell line LP9 and mesothelioma cell lines. (C) PD‐L1 mRNA expression in human normal mesothelial cell line LP9 and mesothelioma cell lines. (D) YAP mRNA expression in human normal mesothelial cell line LP9 and mesothelioma cell lines. Error bars indicate standard deviations; ***P ≤ .001

Western blotting showed that H2052 and 211H cells had increased PD‐L1 protein expression compared to the other 3 cell lines (H290, MS‐1 and H28). In 5 mesothelioma cell lines (H290, H2052, 211H, MS‐1 and H28), the p‐YAP (S127)/YAP protein expression ratio was low (Figure 1A). GTIIC reporter activity of the Hippo Pathway was 14‐ to 30‐fold significantly higher in 5 mesothelioma cell lines (H290, H2052, 211H, MS‐1 and H28) than in human normal mesothelial cell line LP9 and mesothelioma cell line H2452 cells (*P* < .001) (Figure 1B). Real‐time PCR showed that H2052 and 211H cells had 6‐fold higher PD‐L1 (CD274) mRNA expression than LP‐9, H290, MS‐1, H28 and H2452 cells (*P* < .001) (Figure 1C). Five mesothelioma cells (H290, H2052, 211H, MS‐1 and H28) had significantly increased YAP mRNA expression when compared to LP‐9 and H2452 cells (*P* < .001) (Figure 1D). Measured GTIIC reporter activity, and YAP and PD‐L1 mRNA expression of all 6 mesothelioma cell lines and one NSCLC cell line A549 were normalized by control LP‐9 cells and list in Tables S1, S2 and S3. Cell proliferative rate was examined in 4 mesothelioma cell lines (H2052, 211H, H290 and H2452). The growth rate of cells with high YAP and PD‐L1 expression (H2052 and 211H) was much higher than cells with low PD‐L1 expression (Figure S1B).

Our results indicate that H2052 and 211H cells had increased PD‐L1 protein expression and that PD‐L1 mRNA expression is greater in the 2 cell lines than in other mesothelioma cell lines.

### 3.2 Inhibition of YAP decreased PD‐L1 expression in H2052 and 211H cells

**Figure 2:**
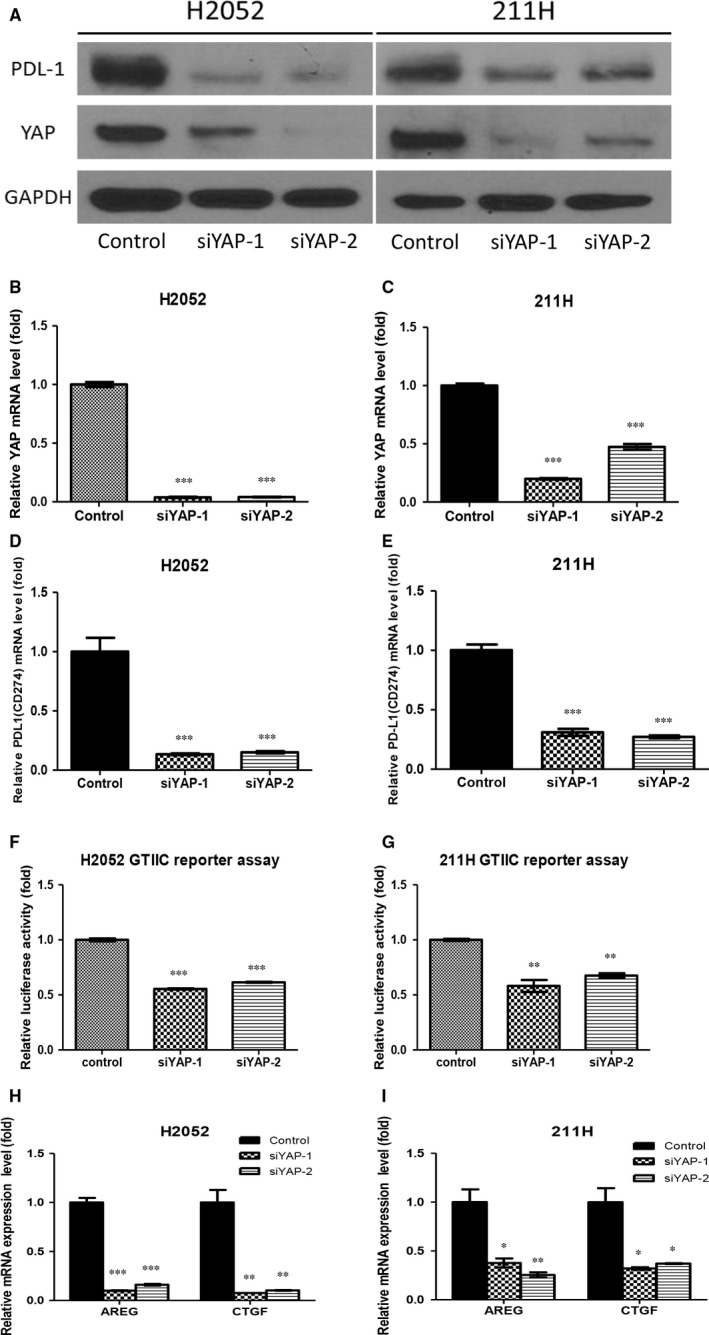
Inhibition of yes‐associated protein (YAP) decreased PD‐L1 expression in mesothelioma H2052 and 211H cells. Western blotting was used to determine PD‐L1 protein expression after YAP knockdown (A). In both cell lines, after YAP knockdown, YAP mRNA expression significantly decreased (B, C), as did PD‐L1 mRNA expression (D, E), and GTIIC reporter activity (F, G). (H) Hippo/YAP downstream gene AREG and CTGF mRNA expression decreased after YAP knockdown in H2052 cells (H) and in 211H cells (I). Error bars indicate standard deviations; *P < .05; **P ≤ .01 ***P ≤ .001

To investigate how YAP inhibition influences PD‐L1 expression, we examined the protein and mRNA levels of PD‐L1 after YAP knockdown in H2052 and 211H cells because these 2 cell lines have increased PD‐L1 protein and mRNA expressions compared to other MPM cell lines. After YAP knockdown by YAP siRNA in both cell lines, PD‐L1 protein expression decreased in both cell lines, as shown by Western blotting (Figure 2A). Real‐time PCR showed that YAP and PD‐L1 mRNA expression also significantly decreased (Figure 2B‐E), as did GTIIC Hippo reporter activity (Figure 2F,G). Finally, transcriptional activities of YAP downstream genes AREG and CTGF were significantly decreased in H2052 and 211H cells after YAP knockdown by YAP siRNA (Figure 2H,I). Our results indicate that YAP knockdown by YAP siRNA decreased PD‐L1 expression at both protein and mRNA levels in mesothelioma H2052 and 211H cells.

### 3.3 Forced overexpression of YAP rescues PD‐L1 expression in YAP down‐regulated H2052 and 211H cells

**Figure 3:**
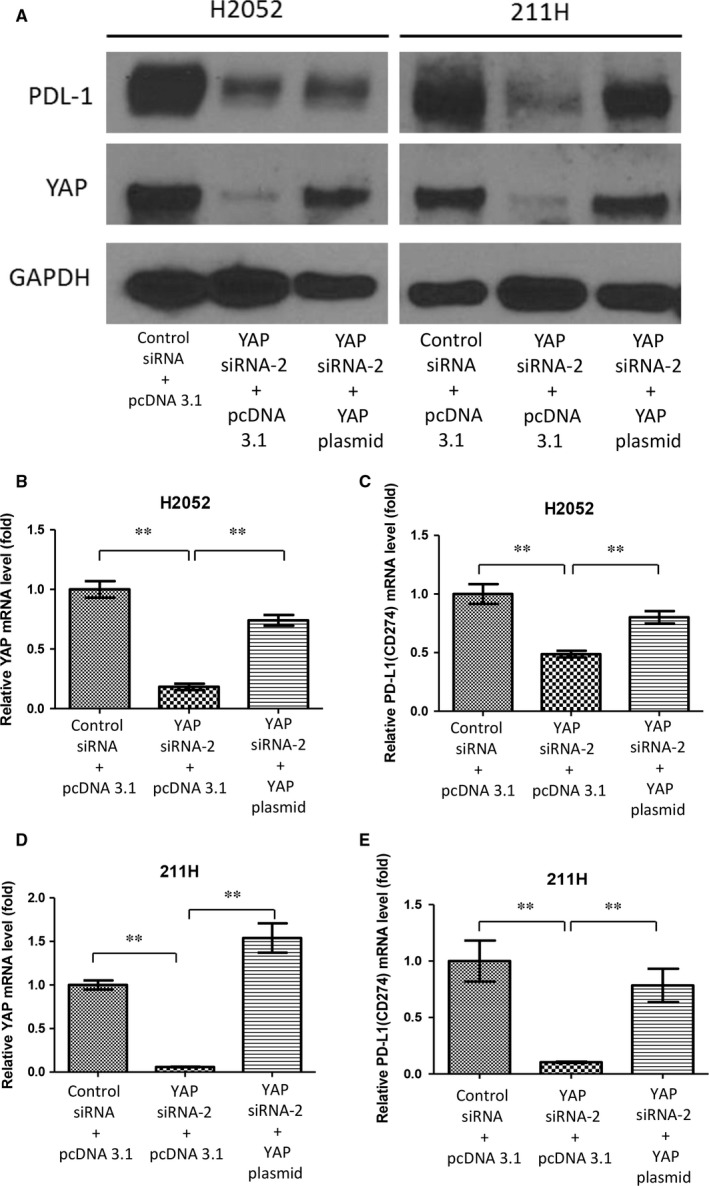
Yes‐associated protein (YAP) forced overexpression rescued PD‐L1 expression during YAP inhibition. After YAP forced overexpression in H2052 and 211H cells treated with YAP 3′UTR siRNA, PD‐L1 protein expression increased in both cell lines, as shown by Western blotting (A), YAP mRNA expression increased significantly in H2052 cells (B) and in 211H cells (D), and PD‐L1 mRNA expression increased significantly in H2052 cells (C) and in 211H cells (E). Error bars indicate standard deviations; **P ≤ .01

To verify that PD‐L1 expression can be regulated by YAP expression, we analysed PD‐L1 expression after YAP inhibition with or without forced overexpression of the YAP gene in H2052 and 211H mesothelioma cells. For this experiment, we used YAP siRNA targeting the 3′UTR end of the YAP gene. In both cell lines, PD‐L1 protein expression decreased after YAP depletion by YAP siRNA. Also in both cell lines, after forced overexpression of the YAP gene, PD‐L1 protein level was increased compared to that in cells treated with YAP 3′UTR siRNA only (Figure 3A). After 3′UTR siRNA treatment, PD‐L1 mRNA expression was significantly decreased compared to that in cells treated with control non‐targeting siRNA. In both cell lines, PD‐L1 mRNA expression was significantly increased after forced overexpression of the YAP gene (*P* < .01) (Figure 3B‐E). Further, we investigated whether forced overexpression of YAP increases PD‐L1 expression in H2452 and A549 cells which have low YAP and low PD‐L1 expression. Western blotting showed that PD‐L1 protein expression increased after YAP overexpression through YAP plasmid transfection in both cell lines (H2452 and A549) (Figure S1C).

These results suggest that PD‐L1 expression is regulated by YAP expression in H2052, 211H, H2452 and A549 cells.

### 3.4 YAP regulates PD‐L1 at the transcriptional level through binding to the enhancers of PD‐L1

**Figure 4:**
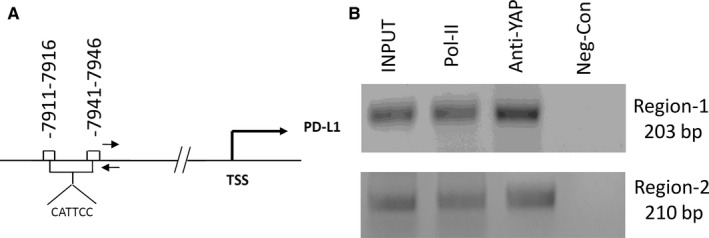
Yes‐associated protein (YAP) binds the enhancers of PD‐L1 and regulates PD‐L1 at the transcriptional level. (A) Sequence analysis of PD‐L1 showed putative YAP binding sites between −7911 and −7946 nucleotides upstream of the transcription start site. (B) ChIP assays were performed with H2052 cells in 2 regions (203 and 210 bp), shown by gel bands of RT‐PCR products

To investigate whether YAP regulates PD‐L1 at the transcriptional level in MPM cells, we examined the PD‐L1 promoter region and found 2 putative TEAD‐binding sites (CATTCC), which are 7911 and 7941 bp upstream of the PD‐L1 transcription start site (Figure 4A). We used chromatin immunoprecipitations (ChIPs) to test our hypothesis in H2052 cells. Fragmented chromatin of H2052 cells was incubated with anti‐IgG (negative control), anti‐YAP and anti‐POL‐II (positive control). We detected the gel bands of RT‐PCR products in 2 regions (203 bp and 210 bp). YAP appeared to bind to the PD‐L1 enhancer in H2052 cells (Figure 4B).

### 3.5 The YAP inhibitor verteporfin decreased PD‐L1 protein and mRNA expression in H2052 and 211H cells

**Figure 5:**
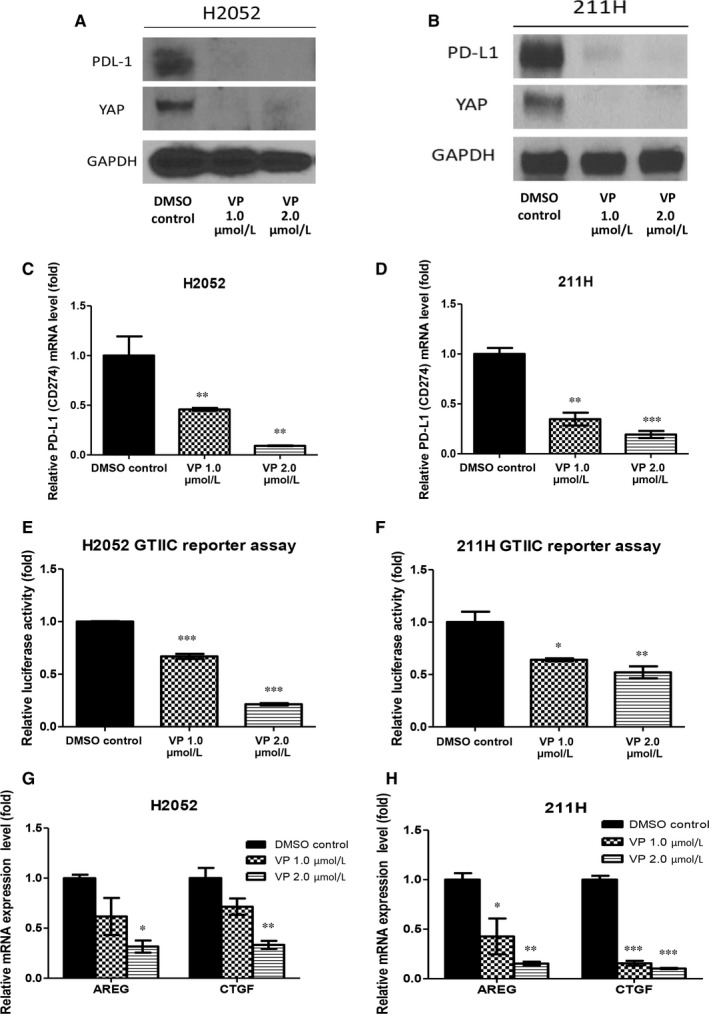
The yes‐associated protein (YAP) inhibitor verteporfin decreased PD‐L1 expression in H2052 and 211H cells. After cells were treated 1.0 and 2.0 μmol/L verteporfin, (A, B) Western blotting showed that PD‐L1 protein expression decreased, (C, D) real‐time PCR showed that PD‐L1 mRNA expression significantly decreased, (E, F) GTIIC reporter activity significantly decreased, and (G, H) Hippo/YAP downstream genes AREG and CTGF significantly decreased. Error bars indicate standard deviations; *P < 0.05; **P ≤ .01 ***P ≤ .001

We next tested whether the YAP inhibitor verteporfin has an effect similar to that of YAP siRNA in inhibiting PD‐L1 expression in H2052 and 211H cells. After 1.0 μM and 2.0 μM verteporfin treatment in H2052 and 211H cells, Western blotting showed that PD‐L1 protein has decreased (Figure 5A,B), real‐time PCR showed that PD‐L1 mRNA expression has significantly decreased (Figure 5C,D), and GTIIC reporter activity of the YAP signalling downstream genes AREG and CTGF significantly decreased (Figure 5E‐H).

### 3.6 Concurrent expression of YAP and PD‐L1 in mesothelioma tumours

**Figure 6:**
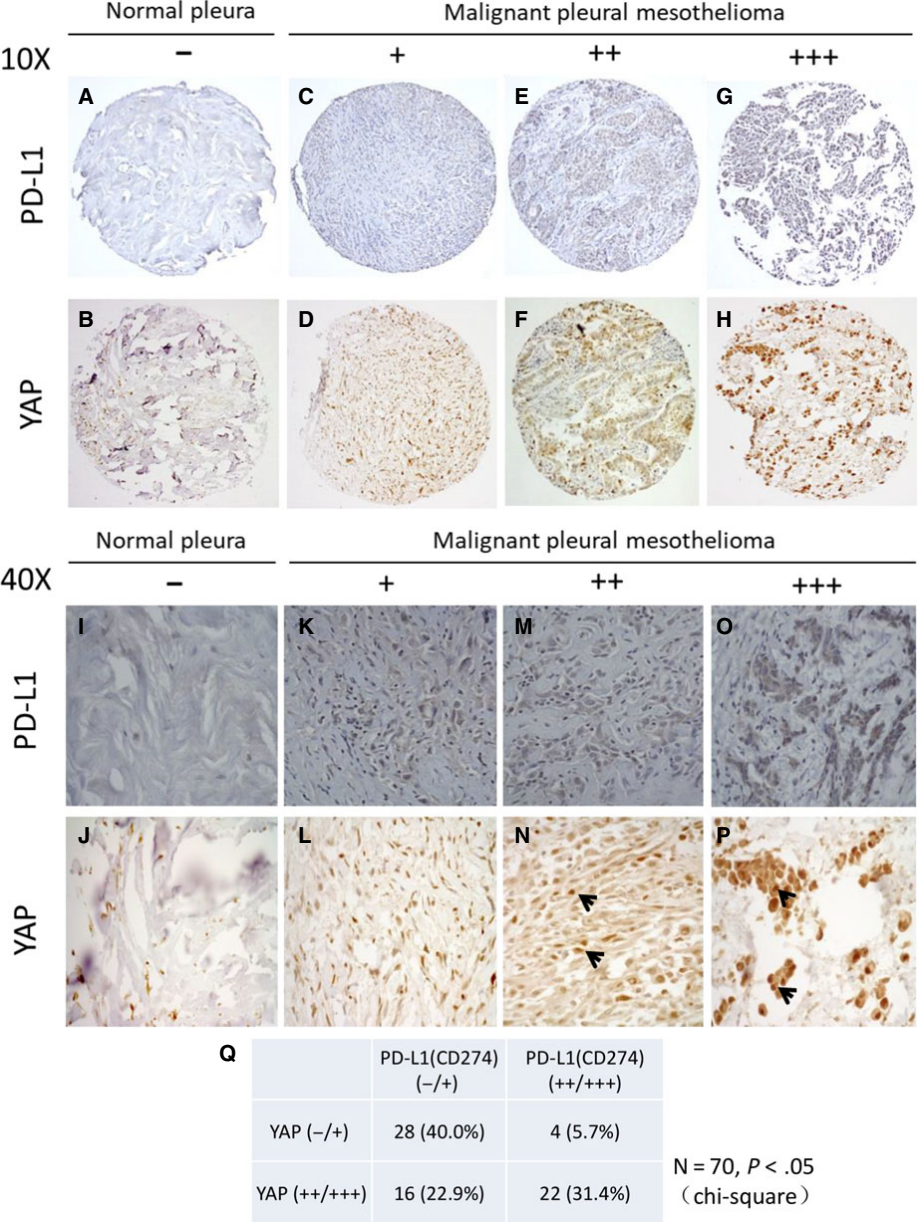
Immunohistochemistry of yes‐associated protein (YAP) and PD‐L1 staining in mesothelioma and normal pleura samples. (A, B, I, J) Normal pleura sample. (C‐H) Mesothelioma samples. (C, D, K, L) Weak PD‐L1 and YAP stain. (E, F, M, N) Moderate PD‐L1 and YAP stain. (G, H, O, P) Strong PD‐L1 and YAP stain. YAP staining localized at nuclei (arrows) of mesothelioma under a 40× objective lens. (Q) Cytoplasmic PD‐L1 staining was concurrent with nucleus YAP staining in mesothelioma samples (*P* < .05, chi‐square test)

We next used immunohistochemistry to investigate the relationship between YAP and PD‐L1 expression in tissue samples from 70 patients with MPM. The YAP and PD‐L1 staining were negative in normal pleural tissues (Figure 6A,B,I,J). PD‐L1 staining in the cytoplasm was negative (−) in 28.6%, weak (+) in 34.2% and moderate to strong (++/+++) in 37.2% (Figure 6C,E,G,K,M,O, Table 1). YAP staining in the nucleus was negative (−) in 15.7% of the samples, weak (+) in 30.0% and moderate to strong (++/+++) in 54.3% (Figure 6D,F,H,L,N,P, Table 1). The relationship between YAP and PD‐L1 was analysed, and we found that moderate to strong PD‐L1 expression was concurrent with moderate and strong nuclear YAP staining (*P* < .05, chi‐square test; Figure 6Q).

**Table 1:** Positive and negative number and ratio of yes‐associated protein (YAP) and PD‐L1 expression in 70 primary mesothelioma samples

	−Number (ratio)	+Number (ratio)	++Number (ratio)	+++Number (ratio)
YAP	11 (15.7%)	21 (30.0%)	23 (32.9%)	15 (21.4%)
PD‐L1	20 (28.6%)	24 (34.2%)	10 (14.3%)	16 (22.9%)

## 4 DISCUSSION

Our study provides several lines of evidence that suggests the YAP signalling pathway regulates PD‐L1 expression in human MPM. First, we found that inhibition of YAP by siRNA decreased PD‐L1 protein and mRNA expression in H2052 and 211H cells. Second, YAP forced overexpression by YAP plasmid transfection rescued PD‐L1 protein and mRNA expression after YAP knockdown in H2052 and 211H cells. Third, ChIPs assay showed that YAP binds to the PD‐L1 enhancer in H2052 cells. Finally, IHC of human mesothelioma tumours showed concurrent PD‐L1 and YAP staining in part of MPM patient samples.

Currently, tumour PD‐L1 expression is used as a predictive biomarker for immunotherapy targeting on the PD1/PD‐L1 axis.^6^ PD‐L1 appears to be expressed in a substantial proportion of human MPM.^12^, ^24^ Our IHC staining results also showed that 22.9% of human MPM had strong expression of PD‐L1, which indicate that some patients with MPM might benefit from anti‐PD‐1/PD‐L1 immunotherapy. YAP and PD‐L1 coexpression in human MPM has not been reported previously. Our study showed this coexpression in 31.4% (22/70) of MPM samples and that the PD‐L1‐positive ratio was higher in moderate to strong nucleus YAP staining samples than in negative to weak nucleus YAP staining samples (*P* < .05, chi‐square). However, these patient samples were initially studied 10‐20 years ago, and there was not enough tissue left to evaluate the further molecular status between YAP and PD‐L1. Although PD‐1/PD‐L1 inhibitors can be a new treatment option for unresectable MPM with high PD‐L1 expression, the efficacy of PD‐1/PD‐L1 inhibitors is under investigation in clinical trials currently.^11^, ^25^, ^26^ After these clinical trials completed and even anti‐PD‐1/PD‐L1 inhibitors are approved in treating patients with advanced MPM, more patient samples should be gathered to investigate the molecular relevance between YAP and PD‐L1 in the future.

The regulation of PD‐L1 in tumours is not well understood. Two recent studies reported that the EGFR pathway is involved in the regulation of PD‐L1 expression in EGFR‐mutant human non‐small‐cell lung cancer (NSCLC) cells, whereby inhibition of EGFR signalling decreased PD‐L1 expression in some EGFR‐mutant NSCLC cells, suggesting that tumour PD‐L1 expression may be regulated by cancer‐driven mutation.^27^, ^28^ Five of the human MPM cell lines (H290, H2052, 211H, H28 and MS‐1) used in our study had a low p‐YAP (S127)/YAP ratio and high GTIIC reporter activity. Two MPM cell lines, H2052 and 211H, showed increased PD‐L1 protein expression and significantly increased PD‐L1 mRNA expression. We previously reported that YAP is frequently activated in human MPM and suggested YAP as a potential therapeutic target.^20^, ^21^ Our current study shows that inhibition of YAP down‐regulates PD‐L1 expression in H2052 and 211H cells. Together, these findings suggest that, in human MPM, YAP can help MPM cells to escape from anticancer immune response by increasing tumour PD‐L1 expression. Our hypothetical model is summarized as Figure S2. Moreover, we recently reported a correlation between YAP and EGFR/extracellular signal‐regulated kinase (ERK) signalling pathways in human NSCLC cells with EGFR mutation.^16^, ^17^ We showed that inhibiting the EGFR/ERK signalling pathway decreased YAP expression in EGFR‐mutant human NSCLC cells. These results may partly explain the recent finding that inhibition of EGFR down‐regulates PD‐L1 expression in EGFR‐mutant NSCLC.^27^, ^28^ EGFR may be involved in regulating PD‐L1 through the activation or inhibition of YAP.

In our MPM tumour microarray samples, some tumours with weak nucleus YAP staining show PD‐L1 expression (5.7%, 4/70). YAP is one of the factors that regulate PD‐L1 expression, and other factors may be involved in regulating PD‐L1 expression in various cancers.^29^, ^30^, ^31^, ^32^ For example, TAZ, another mediator of the Hippo signalling, was reported to contribute to PD‐L1 regulation in human lung cancer cells.^29^

To our knowledge, our study is the first to show that inhibition of YAP down‐regulates PD‐L1 expression in human MPM. Further investigation of how YAP regulates tumour PD‐L1 expression in various cancers is warranted. In the future, treatments that target YAP may be an alternative to immunotherapy targeting on PD‐1/PD‐L1.

## CONFLICT OF INTEREST

All authors have no conflict of interest.

## AUTHOR CONTRIBUTIONS

PC. Hsu and L. You conceived and designed the study. PC. Hsu, J. Miao, Z. Huang, Z. Xu, J. You, and YL. Yang contributed to the development of methodology. PC. Hsu, J. Miao, WQ. Zhang and D.M. Jablons acquired the data (provided human samples, provided facilities, etc.) CW Wang analysed and interpreted the data (pathologic analysis). PC. Hsu, J. Miao, CT.Yang and L.You wrote, reviewed and revised the manuscript. PC. Hsu, J. Miao, WQ. Chang, Z. Xu and L. You contributed to administrative, technical or material support (organizing data). L. You supervised the study.
