# Supplementary figures and images for: Inhibition of yes‐associated protein down‐regulates PD‐L1 (CD274) expression in human malignant pleural mesothelioma
Source: J Cell Mol Med. 2018 Mar 24;22(6):3139–48. doi: 10.1111/jcmm.13593 (PMC5980156; doi:10.1111/jcmm.13593)

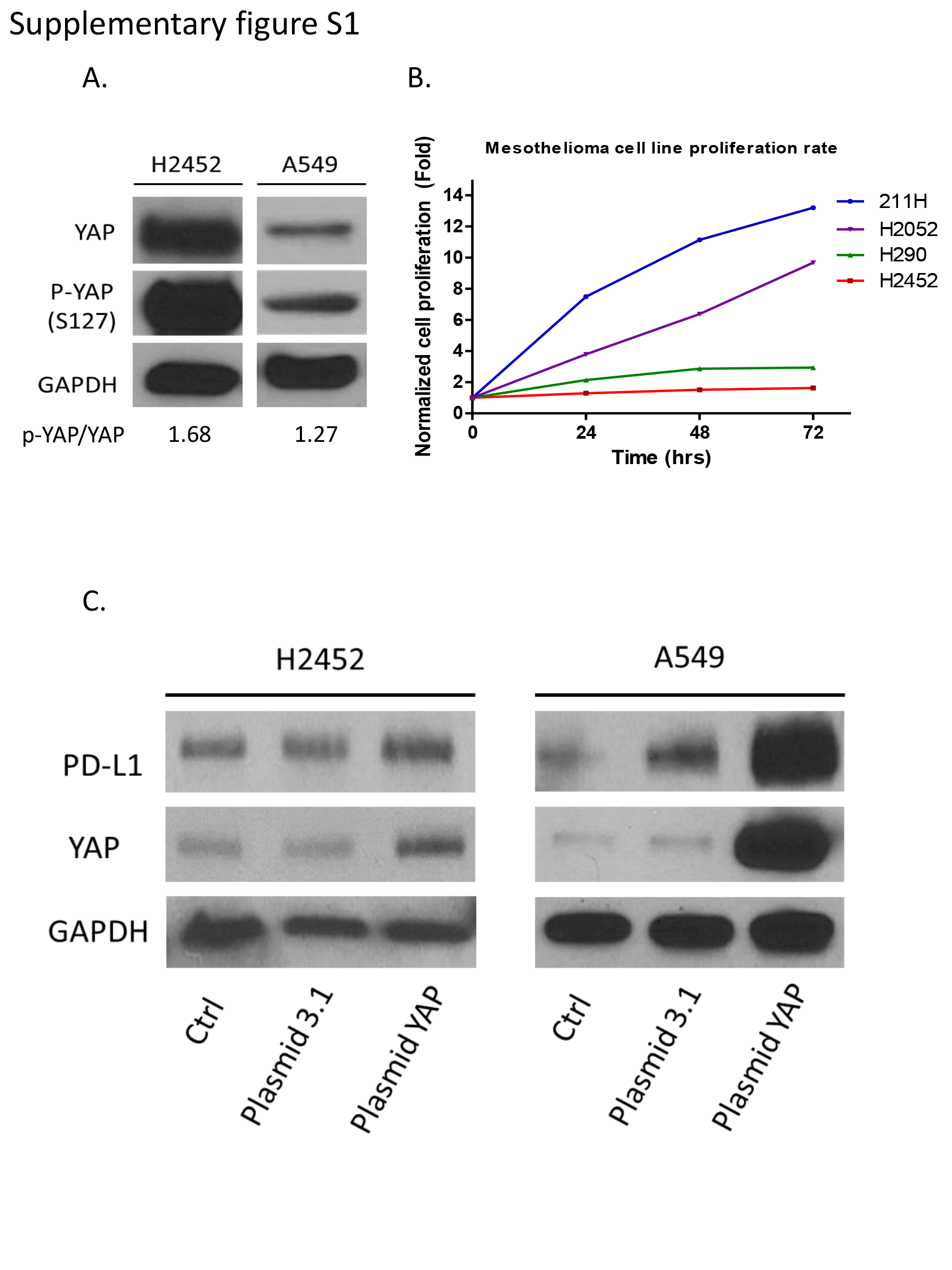

Supplement: Supplementary file 1 [file JCMM-22-3139-s001.tif]

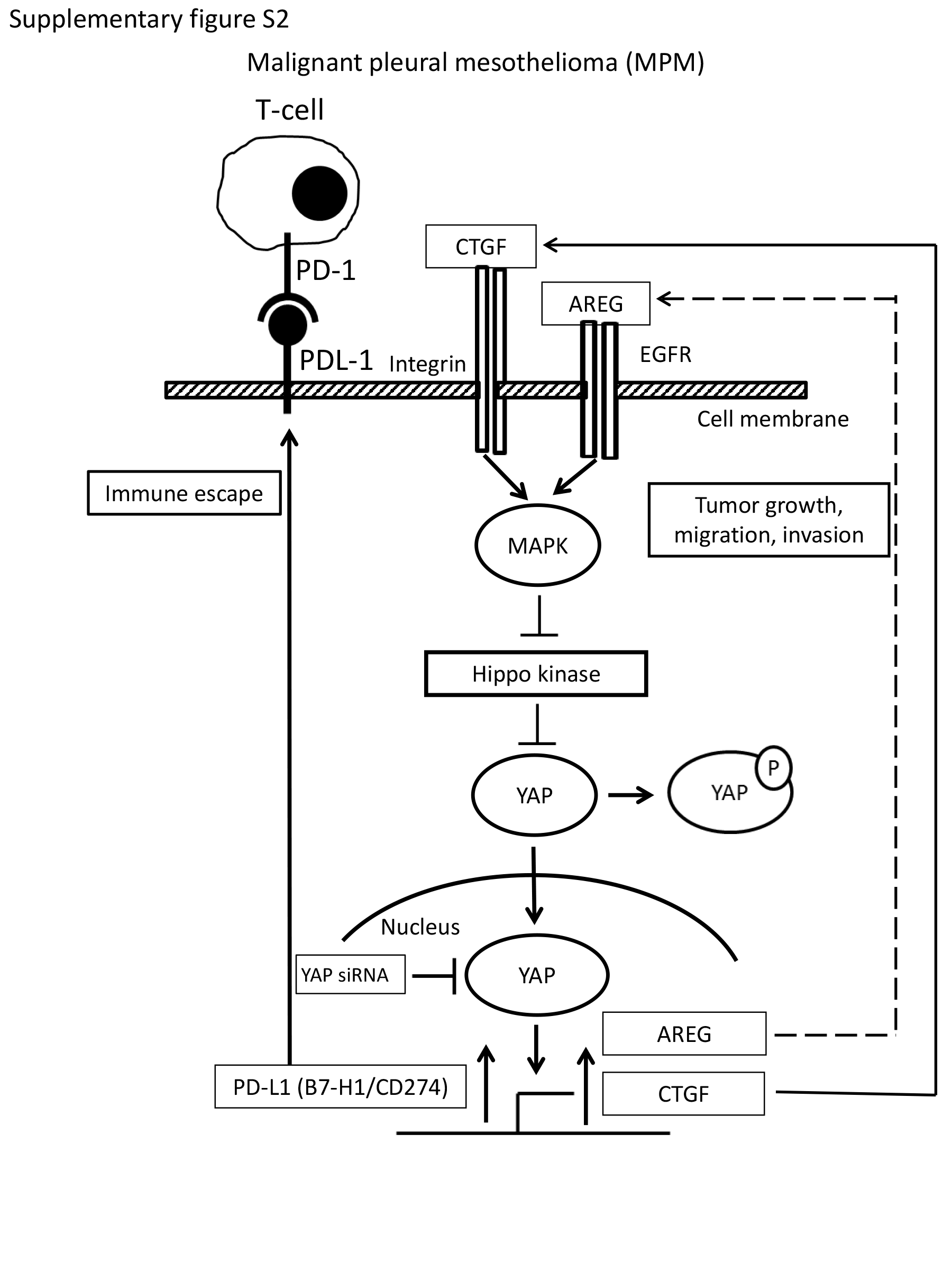

Supplement: Supplementary file 2 [file JCMM-22-3139-s002.tif]
