# Supplementary material for: Inhibition of yes‐associated protein down‐regulates PD‐L1 (CD274) expression in human malignant pleural mesothelioma
Source: J Cell Mol Med. 2018 Mar 24;22(6):3139–48. doi: 10.1111/jcmm.13593 (PMC5980156; doi:10.1111/jcmm.13593)
Supplement: Supplementary file 3 [file JCMM-22-3139-s003.pdf]

Supplementary table S1.

GTIIC reporter activity of MPM cell lines and NSCLC cell line A549

Cell line	Normalized GTIIC reporter activity (Firefly/Renilla) mean ± SD (percentage by control)
LP-9	1.000 ± 0.113
H290	25.215 ± 1.449
H2052	30.626 ± 1.608
211H	24.040 ± 1.695
MS-1	14.868 ± 0.764
H28	22.143 ± 3.102
H2452	0.389 ± 0.117
A549	0.724 ± 0.008
