# Supplementary material for: Inhibition of yes‐associated protein down‐regulates PD‐L1 (CD274) expression in human malignant pleural mesothelioma
Source: J Cell Mol Med. 2018 Mar 24;22(6):3139–48. doi: 10.1111/jcmm.13593 (PMC5980156; doi:10.1111/jcmm.13593)
Supplement: Supplementary file 4 [file JCMM-22-3139-s004.pdf]

Supplementary table S2

YAP mRNA level percentage by control

Cell line	YAP mRNA<br>mean ± SD (percentage by control)
LP-9	1.000 ± 0.041
H290	11.247 ± 1.080
H2052	18.137 ± 2.095
211H	8.663 ± 1.276
MS-1	14.837 ± 3.824
H28	8.209 ± 1.036
H2452	0.799 ± 0.119
A549	0.414 ± 0.085
