# Supplementary material for: Inhibition of yes‐associated protein down‐regulates PD‐L1 (CD274) expression in human malignant pleural mesothelioma
Source: J Cell Mol Med. 2018 Mar 24;22(6):3139–48. doi: 10.1111/jcmm.13593 (PMC5980156; doi:10.1111/jcmm.13593)
Supplement: Supplementary file 5 [file JCMM-22-3139-s005.pdf]

Supplementary table S3.

PD-L1 (CD274) mRNA level percentage by control

Cell line	PD-L1 (CD274) mRNA mean ± SD (percentage by control)
LP-9	1.000 ± 0.192
H290	0.614 ± 0.134
H2052	5.979 ± 0.833
211H	6.281 ± 1.577
MS-1	1.271 ± 0.057
H28	0.382 ± 0.059
H2452	0.468 ± 0.023
A549	0.609 ± 0.091
