# Supplementary material for: Inhibition of yes‐associated protein down‐regulates PD‐L1 (CD274) expression in human malignant pleural mesothelioma
Source: J Cell Mol Med. 2018 Mar 24;22(6):3139–48. doi: 10.1111/jcmm.13593 (PMC5980156; doi:10.1111/jcmm.13593)
Supplement: Supplementary file 6 [file JCMM-22-3139-s006.docx]

**Supplementary figure S1.** (A) YAP, p-YAP (S127) protein expression and p-YAP (S127)/YAP ratio in H2452 and A549 cells. (B) Cell proliferative rate of mesothelioma cell lines, H2052, 211H, H290 and H2452. The growth rate of H2052 and 211H cells (high YAP and high PD-L1 expression) was much higher than H290 (high YAP and low PD-L1 expression) and H2452 (low YAP and low PD-L1 expression cells. (C) YAP forced overexpression increased PD-L1 protein expression in H2452 and A549 cells.

**Supplementary figure S2.** Schematic diagram showing that in some human malignant pleural mesothelioma, YAP enters nucleus and activates PD-L1 expression at transcription level. Increased PD-L1 mRNA expression increases PD-L1 expression on mesothelioma tumour cell surface and helps mesothelioma tumours to escape from anticancer immune response. Inhibition of YAP by siRNA down-regulates PD-L1 expression in human malignant pleural mesothelioma.

**Supplementary table S1** Measured GTIIC reporter activity in human MPM cell lines and NSCLC cell line A549.

**Supplementary table S2** Measured YAP mRNA expression in human MPM cell lines and NSCLC cell line A549.

**Supplementary table S3** Immunohistochemistry of mesothelioma samples with moderate to strong YAP nuclear staining and low PD-L1 expression. The PD-L1 staining is negative to weak either at membrane or cytoplasm.
